# Comparison of Adding Sildenafil Versus Estradiol to Clomiphene Citrate on the Applebaum Score and Pregnancy Rate in Patients With Unexplained Infertility: A Double-Blind Randomized Controlled Trial

**DOI:** 10.7759/cureus.63414

**Published:** 2024-06-28

**Authors:** Jean-Didier Bosenge-Nguma, Antoine Modia O'yandjo, Roland Marini Djang'eing'a, Juakali SKV, Noël Labama Otuli, Alexis Heng Boon Chin, Gédéon Katenga Bosunga

**Affiliations:** 1 Department of Obstetrics and Gynaecology, Faculty of Medicine and Pharmacy, University of Kisangani, Kisangani, COD; 2 Pharmaceutical Analytical Chemistry Laboratory and Interdisciplinary Drug Research Centre, University of Liège, Liège, BEL; 3 Reproductive Medicine, Singapore Fertility and IVF Consultancy Pvt Ltd, Singapore, SGP

**Keywords:** unexplained infertility, applebaum score, estradiol valerate, sildenafil, clomiphene

## Abstract

Background: Both sildenafil and estradiol are seen to improve endometrial thickness in patients with infertility who are undergoing clomiphene induction cycles. However, the correlation between endometrial thickness and pregnancy rate is debatable. This study investigated the effect of adding oral sildenafil to clomiphene citrate (CC), compared to adding estradiol valerate, on the uterine biophysical profile (Applebaum score) and pregnancy rate.

Methods: This was a double-blinded, randomized controlled trial conducted in Kisangani in the Democratic Republic of the Congo from October 1, 2021, to October 31, 2023. Patients with unexplained infertility were randomly assigned to one of two groups: the interventional, which was given CC (2 x 50 mg/day from day 3 to day 7 of the menstrual cycle) + sildenafil (2 x 25 mg/day orally from day 8 to day 12) or (ii) the control group, which was given CC (similar dosage as the intervention group) + EV (2 x 2 mg/day orally from day 8 to day 12), for a maximum of three cycles. Applebaum scores and clinical pregnancy rates were measured.

Results: Patients in the sildenafil and EV groups were similar in mean age (29.04 versus 28.89 years). Of the 74 patients enrolled in each group, 71 in the sildenafil group and 72 in the EV group received treatment and were followed to completion. The Applebaum scores were significantly higher in the sildenafil group than in the EV group (17.05 versus 15.14, respectively, P=0.000). In the sildenafil group, the clinical pregnancy rate was also significantly higher, at 28.92% versus 20.83% in the EV group (P = 0.04).

Conclusion: As compared to EV, the oral addition of sildenafil to CC is associated with a good Applebaum score and a high rate of clinical pregnancy in patients with unexplained infertility.

## Introduction

Clomiphene is a commonly used drug to induce ovulation due to its low cost, tolerability, and safety profile. Although it can achieve good ovarian stimulation and yield high ovulation rates, studies have often reported low pregnancy rates [1,2], as well as high rates of early abortion associated with clomiphene [3]. The anti-estrogenic effects of clomiphene on the endometrium have been identified as factors that may explain the gap between ovulation and pregnancy rates.

The successful implantation of an embryo in the endometrium is crucial for the continuation of pregnancy. Endometrial receptivity is the specific time window during the menstrual cycle when the endometrium acquires the ability to attach, nourish, and sustain the blastocyst. This ability is achieved after the endometrium undergoes several histological changes that optimize its thickness, which is a complex dynamic process regulated by tight coordination between various molecular, cellular, and hormonal mechanisms [4].

The standard clinical practice of most in vitro fertilization (IVF) centres usually involves evaluating the uterus and endometrium before embryo transfer to attain optimal results when the uterus is receptive to embryo implantation. Various markers have been identified for assessing endometrial receptivity, including histological, biochemical, and genetic markers (such as the Endometrial Receptivity Array (ERA)) as well as ultrasound markers [5]. Ultrasound assessment of endometrial receptivity is a practical and widely used non-invasive method in resource-limited countries such as the Democratic Republic of Congo. To improve the results of clomiphene induction cycles, several approaches have been proposed, including the adjuvant use of sildenafil due to the anti-estrogenic effects of clomiphene on the endometrium [6].

Several studies have investigated the effects of sildenafil on endometrial receptivity. Most of these studies have focused on the effect of the drug in preparing the endometrium for embryo transfer and have reported an increase in endometrial thickness following intravaginal administration of sildenafil [7,8]. However, there is also a substantial body of medical literature on the adjuvant use of sildenafil during clomiphene induction cycles. These studies have typically used ultrasound measurements of endometrial thickness to determine the likelihood of treatment success and used vaginal administration of sildenafil.

However, evidence suggests that endometrial thickness may not have a significant correlation with clinical pregnancy rates [9,10]. Instead, some studies suggest that endometrial vascularization, rather than thickness, may be a critical parameter for predicting the chances of implantation in IVF cycles [11]. Under these conditions, an ideal endpoint would incorporate several parameters, including endometrial thickness and vascularization. In addition, depending on the number of doses taken daily and the duration of treatment, vaginal use of sildenafil tablets may be associated with discomfort and local irritation [12]. This study aimed to investigate the effect of adding oral sildenafil to clomiphene citrate (CC), compared to adding estradiol valerate (EV), on the uterine biophysical profile (Applebaum score) and pregnancy rate in patients with unexplained infertility.

This article was previously posted to the Research Square preprint server on February 15, 2024.

## Materials and methods

Study design

This was a double-blind, randomized controlled trial conducted in two health facilities in the city of Kisangani (Cliniques Universitaires de Kisangani and Clinique des Anges de Kisangani) located within the province of Tshopo, northern DRC, from October 1, 2021, to October 31, 2023. The study was approved by the Ethics Committee of the University of Kisangani (approval number: UNIKIS/CER/08/2021). The study was conducted in accordance with the declaration of Helsinki. Patients were provided with explanations of the study's objectives and procedures and provided written informed consent prior to participation. The trial is registered in the Pan African Clinical Trials Registry (registration number: PACTR202310849449401).

Infertile couples underwent a clinical evaluation and standard paraclinical workup to identify the etiological factors of infertility before inclusion in the study. The paraclinical workup included ultrasounds, hormone and tubal patency tests for the female partner, and a spermiogram for the male partner. Additional para-clinical examinations were ordered based on each patient's clinical condition and case parameters. If the couple's evaluation did not reveal an apparent cause of infertility, they were deemed infertile due to unexplained causes. The study protocol was thoroughly explained to the participants, and written informed consent was obtained.

Sampling and selection criteria

The sample size was calculated based on endometrial thickness, which is a reliable indicator of endometrial receptivity. The study considered the findings of Ali Dawood et al. [13] and Fetih et al. [14], who reported an improvement in endometrial thickness with the use of estradiol and sildenafil, respectively. The sample size formula of Kirkwood et al. was applied based on estimating the difference in means between two independent samples [15]. Assuming a 10% loss due to follow-up rate, the study aimed to recruit 148 patients who met the following inclusion criteria: age between 19 and 35 years old, body mass index (BMI) between 18.5 and 29.9 kg/m^2^, regular menstrual cycle lasting between 21 and 35 days, good ovarian reserve, bilateral tubal patency, and a spouse with a normal spermiogram according to WHO standards [16]. Good ovarian reserve was defined by the number of antral follicles per ovary, which ranged from 9 to 24 [17]. Exclusion criteria for participation in this study included the presence of infertility due to an etiological factor in one or both spouses, recent hormonal treatment within the past six months, contraindications to drugs utilized in this study, history of cardiac, renal, hepatic, metabolic or neurological diseases, and refusal to participate.

Randomization and intervention

A total of 148 patients with unexplained infertility, who met the inclusion criteria, were included in the study. Each participant enrolled in the study was given a single code. Participants were then randomized and received one of the two study regimens. Randomization was performed using the permuted block of four technique, based on sequences generated by an independent statistician. The drugs were dispensed using closed envelopes, each containing medication for three cycles of ovarian stimulation. In the intervention group, patients were administered CC (Clomid®, Doppel Farmaceutici S.r.l., Italy) orally at a dose of 2 x 50 mg/day for five days, starting on day 3 of the menstrual cycle. Additionally, sildenafil citrate (Penegra®, Zydus Healthcare Ltd, India) was administered orally at a dose of 2 x 25 mg/day for five days, from day 8 to day 12 of the same menstrual cycle. In the control group, patients were administered CC (Clomid®, Doppel Farmaceutici S.r.l., Italy) orally at a dose of 2 x 50 mg/day for five days, starting on day 3 of the menstrual cycle, and estradiol valerate (Progynova®, Zydus Healthcare Ltd, India) tablet orally at a dose of 2 x 2 mg/day, from day 8 to day 12 of the same menstrual cycle. Participants received their medication at the hospital from the nurse in charge. All drugs utilized in this study underwent quality control at the Laboratory for Analysis and Control of Medicines and Foodstuffs (LACOMEDA), University of Kinshasa, DRC.

Discontinuation of treatment before the third cycle was indicated in the following cases: a positive pregnancy test, a major adverse event, non-compliance with the drug administration protocol, or withdrawal of consent from the study. During treatment, patients received transvaginal ultrasound monitoring of follicular growth every 24 to 48 hours from day 10 of their menstrual cycle until ovulation was confirmed. Ovulation was induced by administering 5000 IU of chorionic gonadotropin (HUCOG®-5000HP, Bharat Serums and Vaccines Limited, India) intramuscularly once until at least one follicle had reached 18 mm in diameter.

The couple was advised to have timed intercourses. Before administering chorionic gonadotropin on the day of ovulation induction, transvaginal ultrasound was used to assess the biophysical profile of the uterus and calculate the Applebaum score.

All ultrasound scans were performed by the same operator using the EDAN Acclarix AX8, version 1.2X software (Edan Instruments, Inc., Shenzhen, China). The uterine biophysical profile parameters assessed were endometrial thickness, endometrial stratification, endometrial zone 3 blood flow, myometrial echogenicity, uterine artery Doppler flow (assessed by pulsatility index (PI)), myometrial blood flow, and myometrial contractions.

The endometrial thickness, measured in mm, was determined on the longitudinal section of the uterus by measuring the maximum distance between each myometrial/endometrial interface. Endometrial thickness <7 mm was scored as 0, thickness 7-9 mm was scored as 2, thickness 10-14 mm was scored as 3, and thickness >14 mm was scored as 1 [5]. Endometrial stratification was assessed on the longitudinal section, and one of three modalities was recorded: no stratification, blurred appearance with five lines, or appearance with five distinct lines. Unstratified endometrium was scored 0, stratified but blurred endometrium was scored 1, and stratified endometrium with five distinct lines was scored 3. The study analyzed myometrial echogenicity on a longitudinal scan of the uterus, classifying it as either coarse/inhomogeneous or completely homogeneous. Inhomogeneous myometrium was given a score of 1, while homogeneous myometrium was given a score of 2. Additionally, the presence or absence of blood flow in zone 3 of the endometrium was noted. Absence of blood flow was scored 0, present but sparse blood flow was scored 2, and multifocal flow was scored 5. 

Uterine Doppler was performed on the ascending branch of the uterine arteries, in their segments located at the same level as the internal os of the cervix, with automatic PI calculation. PI ≥ 2.5 was assigned a score of 0, PI from 2.2 to 2.49 was assigned a score of 1, and PI < 2.19 was assigned a score of 2. Myometrial blood flow was measured within the arched vessel, and the presence, absence, or reversal of blood flow at the end of diastole was recorded. The absence of myometrial blood flow was a score of 0 and the presence of myometrial blood flow was a score of 1. Myometrial contraction was observed as endometrial movement, and these contractions propagate from the internal cervical os to the fundus of the uterus during the late follicular phase. Detection of three or more contractions during the two-minute period corresponded to a score of 3, while fewer than three contractions during the same period corresponded to a score of 0 [5]. The Applebaum score was the sum of the scores for the different parameters of the uterine biophysical profile. The score ranges from 0 to 20. The various values found were divided into four categories: ≤13, 14-16, 17-19 and 20. An Applebaum score of 20 was considered perfect, while a score of ≤13 was considered unfavorable [5].

Two weeks after ovulation induction, a serum beta-human chorionic gonadotropin (β-hCG) titer was conducted to confirm pregnancy. Patients with a positive test were scheduled appointments for clinical pregnancy ultrasound confirmation. Individuals with a negative test result resumed a new stimulation cycle on day 3 of their menstrual cycle, using the same drug protocol, for a maximum of three consecutive cycles.

Expected outcomes

The study's primary outcome was the pregnancy rate, determined by the occurrence of pregnancies. Clinical pregnancy was defined as the visualization of one or more gestational sacs with ultrasound scans, whether normal or ectopic [18]. The secondary outcome was the determination of the Applebaum score.

Statistical analysis

The study results are presented as frequencies, percentages, and means ± standard deviations (SD). The primary and secondary outcomes were assessed based on the number of ovarian stimulation cycles. The main objective was to compare the clinical pregnancy rates between the two treatment protocols used in the study. The pregnancy rate was defined as the number of pregnancies per 100 stimulation cycles [18].

The secondary objective of the study was to compare the biophysical profile between the two randomization groups, by determining the Applebaum score. The parameters of the Applebaum score, including endometrial thickness, endometrial stratification, endometrial zone 3 blood flow, myometrial echogenicity, uterine artery Doppler flow assessed by PI, myometrial blood flow, and myometrial contractions, were determined based on the number of stimulation cycles.

To compare proportions, we calculated Pearson's *χ*^2^ test with a significance level of P < 0.05. For continuous variables, we used the student’s t-test, and for categorical variables, we measured the strength of association using the relative risk (RR) with its 95% confidence interval (CI). We performed all analyses using the EPI Info^TM^ software version 7.2.2.6 (Centers for Disease Control and Prevention, Atlanta, Georgia, United States).

## Results

Enrolment of participants

**Figure 1:**
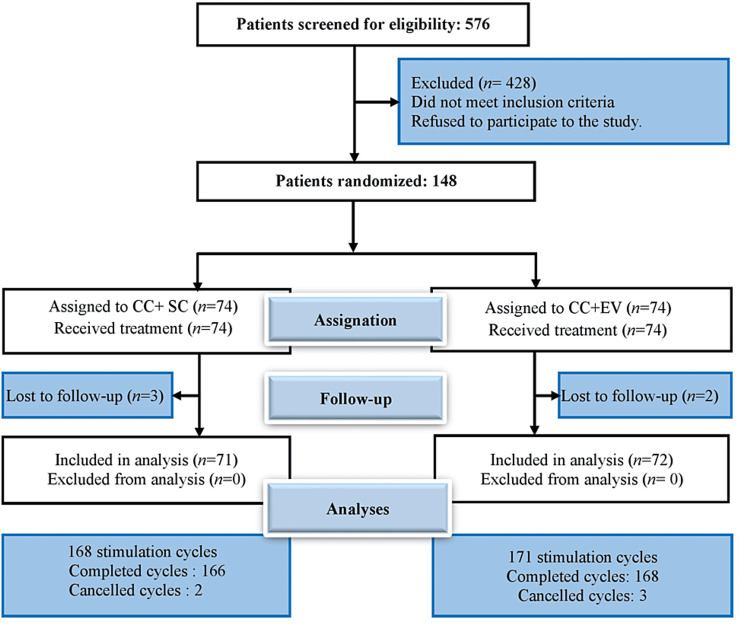
Flowchart depicting patient enrollment, randomization, follow-up, and analysis of results. CC: clomiphene citrate; SC: sildenafil citrate; EV: estradiol valerate

During the study period, a total of 576 patients with infertility were examined in the two care facilities that were used as study settings. Out of these, 428 were excluded because they did not meet the inclusion criteria or refused to participate in the study. The remaining 148 patients were randomized into two groups: 74 participants were assigned to clomiphene + sildenafil treatment (intervention group), and the other 74 were assigned to clomiphene + estradiol valerate treatment (control group). During the follow-up period, three patients from the intervention group and two patients from the control group were lost to follow-up and withdrawn from the study (Figure 1). The sildenafil group underwent a total of 168 stimulated cycles, while the control group underwent 171 cycles.

Baseline patient characteristics at randomization

The mean age of patients in the intervention group was 29.04 years, which was comparable to the control group's mean age of 28.89 years. Both groups had a mean attempting-to-conceive duration of 4.3 years. Table 1 illustrates that the two randomization groups were similar in terms of sociodemographic and clinical characteristics.

**Table 1:** Baseline patient characteristics at randomization CC: clomiphene citrate; SC: sildenafil citrate; EV: estradiol valerate; SD=standard deviation *P* *value (chi-squared test)

Parameters	Categorization	CC + SC (N=71)	CC + EV (N=72)	Student's t-test
Age	Mean ± SD	29.04±2.57	28.89±2.54	0.720
	< 30 years old, n (%)	45 (63.38)	45 (62.50)	
	≥ 30 years old, n (%)	26 (36.62)	27 (37.50)	
Duration of attempt at conception	Mean ± SD	4.39±1.98	4.36±2.01	0.920
	≤ 2 years, n (%)	14 (19.72)	16 (22.22)	
	> 2 years, n (%)	33 (46.48)	30 (41.67)	
	> 5 years, n (%)	24 (33.80)	26 (36.11)	
Type of infertility	Primary, n (%)	26 (36.62)	25 (34.72)	0.408*
	Secondary, n (%)	45 (63.38)	47 (65.28)	
Parity	Mean ± SD	0.94±1.30	0.87±1.26	0.750
	Nulliparous, n (%)	39 (54.93)	42 (58.33)	
	≥ 1, n (%)	32 (45.07)	30 (41.67)	
Body mass index	Mean ± SD	22.76 ± 1.13	22.46 ± 0.96	0.095
Antral follicle count	Mean ± SD	11.51 ± 1.49	11.46 ± 1.23	0.831
Anti-Müllerian hormone	Mean ± SD	2.28 ± 0.47	2.31 ± 0.48	0.686
Prolactin	Mean ± SD	16.42 ± 2.63	15.67 ± 2.79	0.097

Primary outcome (pregnancy rate)

**Figure 2:**
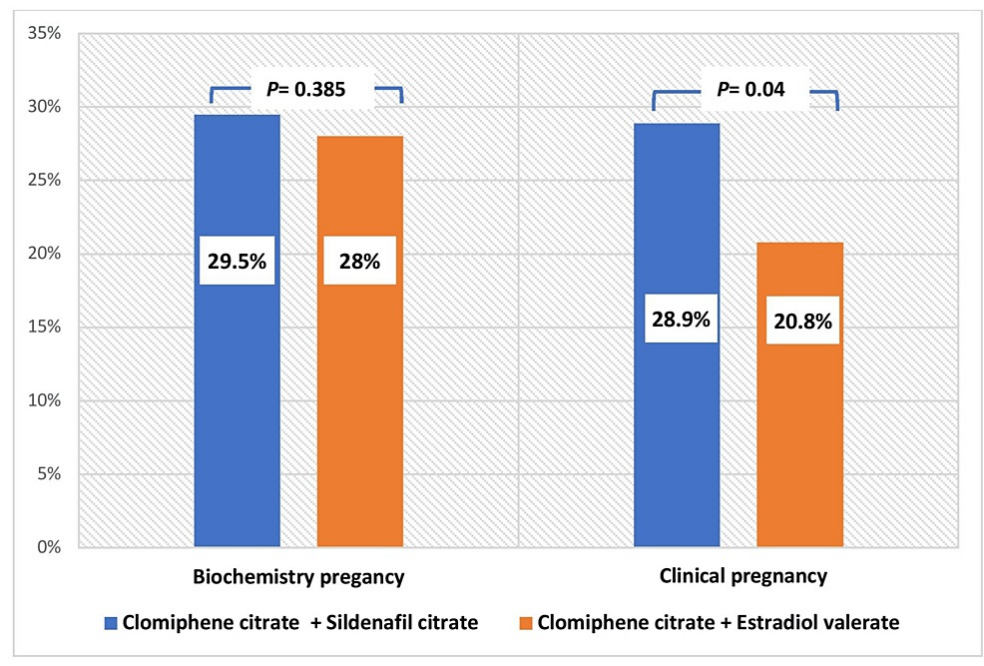
Primary study results (pregnancy rate)

The study's primary results were based on complete ovarian stimulation cycles. Out of the 339 cycles stimulated, 334 (98.5%) were completed cycles, while five cycles (1.5%) were cancelled due to an exaggerated response (more than three mature follicles). Figure 2 displays the primary results of the study. The two randomized groups had a similar biochemical pregnancy rate of 29.52% in the intervention group and 28.05% in the control group (P* *=0.385). However, the clinical pregnancy rate was significantly higher in patients who received sildenafil (28.92%) compared to the control group (20.83%) with a P value of 0.04 (RR = 1.23, 95%CI 0.98-1.54).

Secondary outcomes of the study (Applebaum score)

Table 2 presents the results related to the Applebaum score. The two randomized groups were comparable in terms of endometrial thickness and layering, myometrial echogenicity, blood flow, and contractions. However, the mean Applebaum score was significantly higher in the sildenafil group than in the estradiol group (P = 0.000).

**Table 2:** Applebaum score in the two randomization groups CC: clomiphene citrate; SC: sildenafil citrate; EV: estradiol valerate; SD: standard deviation; NA: not applicable; PI: pulsatility index

Applebaum score parameters	Received treatment		Student's t-test
	CC + SC, mean ± SD	CC+ EV, mean ± SD	
Endometrial thickness	2.55 ± 0.5	2.45 ± 0.55	0.079
Endometrial layering	2.70 ± 0.74	2.68 ± 0.77	0.863
Endometrial blood flow within zone 3	3.99 ± 1.54	2.88 ± 2.65	0.000
Uterine artery Doppler flow evaluated by PI	1.58 ± 0.71	0.89 ± 0.94	0.000
Myometral blood flow	2 ± 0	2 ± 0	NA
Myometral echogenicity	1.94 ± 0.05	1.92 ± 0.07	0.537
Myometral contractions	2.29 ± 1.28	2.30 ± 1.27	0.952
Total	17.05 ± 2.82	15.14 ± 2.90	0.000

Applebaum's uterine scoring system and pregnancy rate

Table 3 shows the pregnancy rate in relation to the Applebaum score. In the sildenafil group, 30.1% of patients had an Applebaum score of 20, compared to 10.7% in the estradiol group. Additionally, we observed a parallel increase in the clinical pregnancy rate with the Applebaum score.

**Table 3:** Relationship between Applebaum's uterine scoring system and pregnancy rate CC: clomiphene citrate; SC: sildenafil citrate; EV: estradiol valerate

	Received treatment		Biochemical pregnancy		Clinical pregnancy	
Score	CC + SC (N=166), n (%)	CC + EV (N=168), n (%)	CC + SC (N=166), n (%)	CC + EV (N=168), n (%)	CC + SC (N=166), n (%)	CC + EV (N=168), n (%)
≤ 13	14 (8.4)	39 (23.2)	0 (0)	1 (0.6)	0 (0)	1 (0.6)
14-16	56 (33.7)	74 (44.1)	12 (7.2)	16 (9.5)	11 (6.6)	11 (6.5)
17-19	46 (27.7)	37 (22)	16 (9.6)	20 (11.9)	17 (10.2)	14 (8.3)
20	50 (30.1)	18 (10.7)	21 (12.7)	9 (5.4)	21 (12.7)	9 (5.4)

## Discussion

Endometrial receptivity is considered a key factor in the successful treatment of infertility [19]. Various methods have been described in the literature to assess it, including the Applebaum score [6]. CC, the drug most commonly prescribed to infertile patients, is known to exert adverse effects on the endometrium. In fact, the use of clomiphene to induce ovulation is often associated with the disparity between high ovulation rates (70-80%) and low pregnancy rates (10-20%) [20].

High rates of subclinical abortion have also been reported following CC induction cycles [21]. The low pregnancy rate with CC induction cycles is attributed to its anti-estrogenic effect on the endometrium and cervical mucus [21,22].

Several studies have been conducted to determine the best method for improving endometrial receptivity and thus pregnancy rate at the end of ovarian induction cycles with CC, with mixed results [8,23-26]. In this study, we found that the addition of sildenafil during clomiphene induction cycles resulted in significantly higher Applebaum scores and clinical pregnancy rates, compared with the use of estradiol valerate, in patients with unexplained infertility. In addition, the clinical pregnancy rate was higher in patients with an Applebaum score of 20, than in those with a lower score.

Similar results have also been reported by Mohamed et al. [27] and Kumar et al. [20]. These studies reported an improvement in endometrial thickness and a higher pregnancy rate with the adjuvant use of sildenafil during CC induction cycles. However, other studies suggest that there is no significant correlation between endometrial thickness and clinical pregnancy rates in IVF cycles [9,28].

Endometrial vascularization, determined by three-dimensional power Doppler ultrasound, has been proposed as a parameter that may have a better predictive value over endometrial morphological appearance for implantation rate in IVF cycles [11]. Other studies recommend the measurement of four different parameters for ultrasound assessment of endometrial receptivity: endometrial thickness, endometrial pattern, endometrial volume (measured by three-dimensional ultrasound) and sub-endometrial blood flow (measured by Doppler ultrasound) [29].

The Applebaum score used in this study includes a total of seven parameters and provides an efficient assessment of endometrial receptivity and a good prediction of the success rate of ovarian stimulation. Malhotra et al. reported that a "perfect Applebaum score" of 20 was associated with a 100% pregnancy rate, while scores of 17-19 were associated with an 80% pregnancy rate, scores of 14-16 were associated with a 60% chance of pregnancy, and scores ≤ 13 resulted in no pregnancy [5]. The same authors also noted that the absence of endometrial flow was always associated with failure to conceive, despite high scores for other parameters.

Study strengths and limitations

One of the strengths of this study is that it compares the effects of the adjuvant use of sildenafil with those of another adjuvant (estradiol valerate). There is extensive literature on the efficacy of sildenafil as an adjuvant in a cycle of ovarian stimulation with CC. However, the comparison has generally been with a placebo [6,16]. Additionally, ultrasound assessment of endometrial receptivity is a non-invasive, convenient, simple, reliable and reproducible method that can be used in resource-limited countries.

Limitations include the small number of patients included in the study. Large-scale studies will allow us to validate the score in our setting. Additionally, the effect of sildenafil citrate on female sexuality was not investigated in this study. However, studies utilizing self-reported measures of sexual functioning yielded inconclusive results. Conversely, studies examining the physiological effects of sildenafil citrate consistently reported significant effects on the various sexual pathways in healthy women, resulting in an improved sexual experience [30]. Further studies are required to ascertain whether an enhanced sexual response represents another potential avenue through which sildenafil can contribute to the success of conventional infertility treatment.

## Conclusions

The adjuvant oral use of sildenafil during ovulation induction cycles with CC is associated with a good Applebaum score and a high clinical pregnancy rate in patients with unexplained infertility, as compared to estradiol valerate. In addition, the system for assessing the biophysical profile of the uterus (Applebaum score) provides a good assessment of endometrial receptivity. In this study, patients in the sildenafil group had significantly good uterine artery PI (P* *= 0.000), good endometrial blood flow (P* *= 0.000), and good total uterine Applebaum scores (P* *= 0.000) compared with those in the estradiol valerate group. Indeed, sildenafil deserves to be used routinely as an adjuvant in patients undergoing CC therapy to improve the success rate.
